# Mixed Reality-Assisted Endoscopic Dacryocystorhinostomy for Post-Traumatic Nasolacrimal Obstruction with Extremely Distorted Maxillofacial Anatomy—Case Report

**DOI:** 10.1177/29941520261441963

**Published:** 2026-04-10

**Authors:** Maja Nowak, Piotr Jakub Gaca, Rafal Nowak

**Affiliations:** 1 Department of Ophthalmology, Jozef Strus City Hospital, Poznan, Poland.; 2 Department of General and Paediatric Ophthalmology, Medical University of Lublin, Lublin, Poland.

**Keywords:** mixed reality, endoscopic DCR, lacrimal, NLDO, SALDO, EnDCR

## Abstract

**Purpose:**

To describe the diagnostic workflow and clinical outcomes of a mixed reality (MR)-assisted endoscopic dacryocystorhinostomy (EnDCR) using the Magic Leap 2 (ML2) headset, performed for a patient with secondary acquired nasolacrimal duct obstruction resulting from extremely distorted post-traumatic maxillofacial anatomy.

**Case Presentation:**

A 40-year-old male with a history of massive maxillofacial trauma 10 years earlier presented to the Department of Ophthalmology of Jozef Strus City Hospital with recurrent dacryocystitis and a dacryocele. Physical examination showed a marked post-traumatic groove over the left orbit, inferior dislocation of the left globe, paralytic strabismus, ptosis, and a large left dacryocele. Nasal endoscopy demonstrated severe bilateral septal deformities obscuring visual access to the nasal cavity. Endoscopic septoplasty was done to obtain surgical access, followed by EnDCR assisted with MR. Preoperative planning included patient-specific 3D prints reconstructed from CT (OsiriX MD^TM^), refined (MeshLab^TM^, Blender^TM^), exported as GLB files, and visualized on ML2; an ML *Spectator* mobile phone app documented the procedure. MR was used preoperatively and intraoperatively as a non-dynamic 3D *image reference* tool (no instrument tracking) to enable the surgeon to maintain a consistent spatial orientation for the lacrimal sac fossa and adjacent bones during septoplasty and EnDCR. Over 9 months post-operation, the patient reported no symptoms (Munk score: 0), lacrimal irrigation confirmed a widely patent ostium, and no further dacryocystitis occurred.

**Conclusions:**

This case supports the use of MR in preoperative and intraoperative planning in extremely complex anatomical distortions.

## Introduction

Mixed reality (MR), a subdomain within medical extended reality (MXR), enables surgeons to visualize three-dimensional (3D) holograms of patient-specific anatomy in real space through head-mounted displays (HMDs). A recent study proposed that MR-assisted surgery can be deployed along a spectrum ranging from (i) image reference (MR-IR; static 3D models positioned in operating room space), through (ii) image guidance (registered overlays without instrument tracking) to (iii) instrument-tracked surgical navigation.^1^ Early evaluations across surgical fields suggest promise for improved spatial understanding and workflow efficiency, while underscoring methodological and regulatory evaluation challenges for safety and effectiveness.^2^

In the field of MXR, numerous studies have demonstrated the advantages of preoperative surgical planning using virtual reality (VR).^3^ Clinical intraoperative experience with MR is increasingly reported across general and visceral surgery, spine surgery, orthopedics, and maxillofacial surgery, demonstrating feasibility and high placement accuracy, particularly when tracked navigation is available. However, the observed benefits depend on the class of device, the accuracy of registration, and the specific clinical task.^1,4,5^ At the field level, emerging taxonomies and guidelines are standardizing MXR terminology and use cases to support rigorous reporting and translational adoption.^2^

Complex post-traumatic nasolacrimal duct obstruction (NLDO) presents formidable challenges for endoscopic dacryocystorhinostomy (EnDCR): displaced lacrimal sac fossa, distorted skull anatomy, and obliterated intranasal landmarks often preclude straightforward access and increase the risk of misdirected osteotomy. Recently, MR-assisted EnDCR approaches have been reported for syndromic and traumatic lacrimal disease, demonstrating feasibility in profoundly altered anatomy.^6^ Here, we report an EnDCR approach assisted intraoperatively by MR used strictly as *image reference* via an HMD to maintain spatial orientation in a patient with severe post-traumatic NLDO complicated by massive septal deviation.

## Case Presentation

### Patient and history

A 40-year-old male was referred to the Department of Ophthalmology at Jozef Strus City Hospital in Poznan, Poland, for dacryocystorhinostomy surgery because of a left-side dacryocele present for 7 years and 13 episodes of dacryocystitis over 10 years following massive maxillofacial trauma. He reported persistent epiphora, recurrent painful swelling at the medial canthus, and periodic purulent discharge treated with antibiotics.

### Examination

Dacryological and ophthalmical evaluation showed a large left dacryocele, a significant post-traumatic groove over the left orbit, severely impaired vision of the left eye, inferior dislocation of the left globe, paralytic strabismus, and ptosis. Nasal endoscopy revealed severe bilateral post-traumatic septal deviation without visual access behind the anterior nares. The working diagnosis was left-side post-traumatic NLDO with dacryocele and massive septal deviation.

### Imaging

CT confirmed post-traumatic skull deformities with displacement of the lacrimal sac fossa, conchae, and nasolacrimal canal and the presence of a dacryocele.

### Ethics and consent

Written informed consent was obtained from the patient for participation in this study and for the publication of clinical details and images. The study adhered to the principles of the Declaration of Helsinki and received approval from the Bioethics Committee of the Regional Medical Council (Resolution No. 33/2025) on 19 March 2025.

## Mixed Reality Setup and Workflow

### Preoperative modeling

The CT DICOM dataset was segmented (OsiriX MD^TM^, Pixmeo SARL, Bernex, Switzerland), mesh-cleaned and decimated (MeshLab^TM^, Visual Computing Lab, ISTI-CNR, Pisa, Italy), and finalized in Blender^TM^ (Blender Foundation, Amsterdam, the Netherlands). The composite model (including skull, lacrimal sac, nasal cavity surface, and septal deformities) was exported to a GLB file.

### Hardware and deployment

The Magic Leap^TM^ 2 (ML2; Magic Leap Inc., Plantation, FL, U.S.A.) HMD was used as the intraoperative MR-IR device. ML2 and HoloLens^TM^ (Microsoft, Redmond, WA, U.S.A.) are one of the few HMDs with a see-through display, where the user can see the surrounding space directly through transparent lenses. 3D objects are displayed in the visual field as holograms. After digital processing of CT data, the GLB model was uploaded to ML2. A companion mobile phone with the ML Spectator app (Magic Leap Inc., Plantation, FL, U.S.A.) documented a third-person view of the surgeon-hologram interaction. MR was used only as *image reference* (MR-IR): the hologram was anchored in the operating room space beside an endoscopy display and manipulated as needed. No instrument tracking or overlay registration on the patient’s face was used.

### Rationale for MR mode selection

In this case, severe intranasal distortion impaired standard spatial cues. MR-IR was selected to provide an always-available, patient-specific 3D “atlas” of the individual’s altered maxillofacial anatomy, complementing endoscopic views without imposing navigational registration constraints. The standard surgical navigation system was unavailable. Similar MR use patterns (heads-up visual support without instrument tracking) have been reported to improve spatial awareness and surgeon confidence in complex anatomy across surgical domains.^1,5–11^

## Surgical Procedure

### Anesthesia and setup

**FIG. 1.**
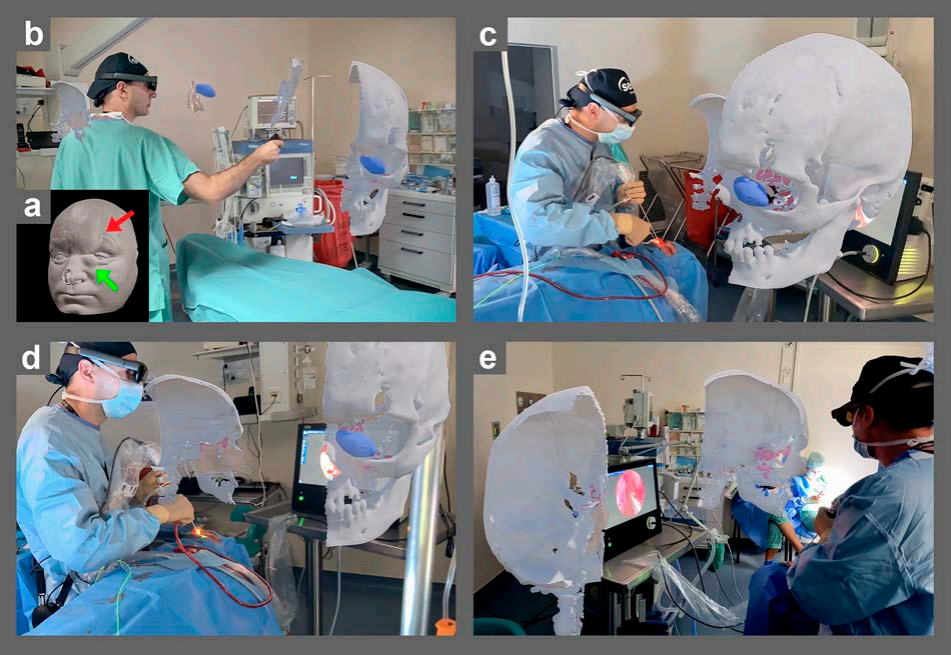
MR-supported operation theatre. **(a)** face 3D reconstruction obtained from CT (red arrow-groove after maxillofacial trauma, green arrow—massively enlarged lacrimal sac; left globe dislocated inferiorly; left ptosis); **(b)** operation theatre setup for MR-supported surgery; **(c–e)** MR-assisted EnDCR.

The procedure was performed under general anesthesia using a standard endoscopy unit (TELE PACK+, Karl Storz SE & Co. KG, Tuttlingen, Germany). An ENT unit (XPS Nexus, Medtronic, Minneapolis, MN, U.S.A.) was used for EnDCR. The surgeon initially positioned the holographic reconstructions in his field of view via ML2 before surgery and subsequently adjusted their position after the patient was induced under general anesthesia (Fig. 1).

### Step 1: Endoscopic septoplasty for access

**FIG. 2.**
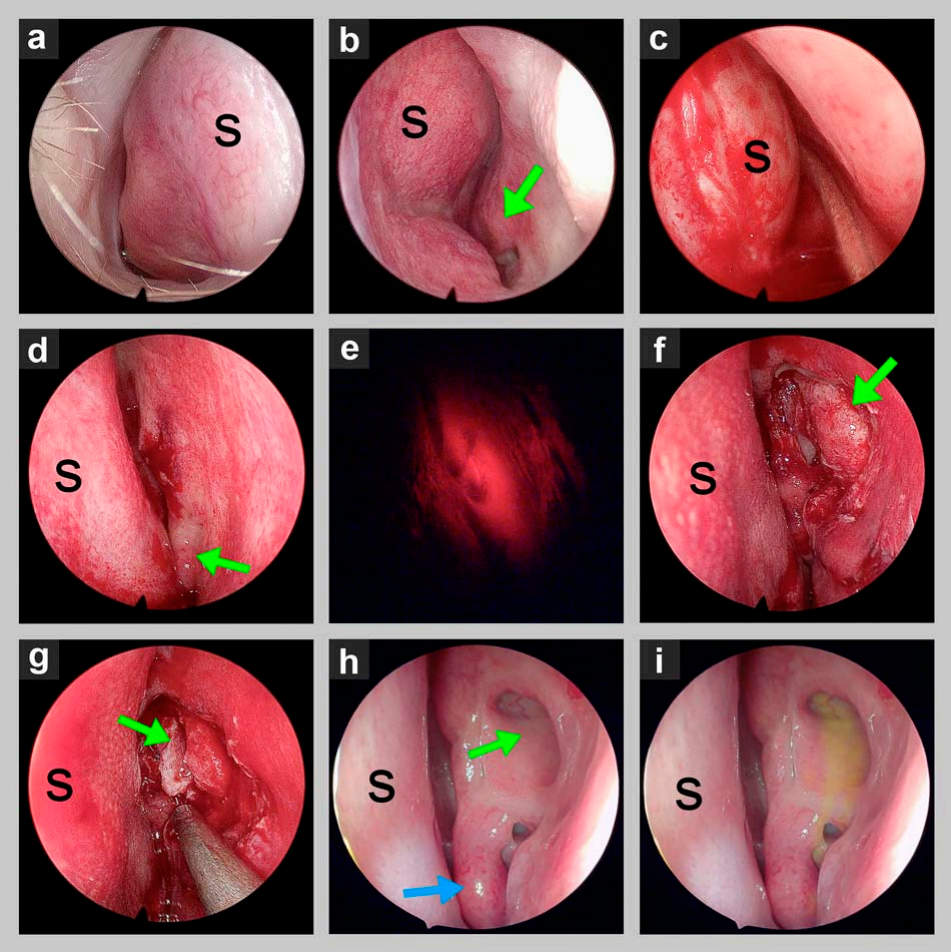
Endoscopic DCR procedure. **(a)** right nasal cavity, massive septal deviation, S-septum; **(b)** left nasal cavity, massive septal deviation, green arrow- inferior turbinate, middle turbinate not visible, S-septum; **(c)** septoplasty: dissected mucosa from the septum, S-septal cartilage; **(d)** left nasal cavity after septoplasty, green arrow—hypotrophic middle turbinate dislocated inferiorly; **(e)** left nasal cavity: light guidance to locate the position of the lacrimal sac; **(f)** left nasal cavity: exposed lacrimal sac located over the middle turbinate, S-septum; **(g)** left nasal cavity: green arrow- incised lacrimal sac, S-septum; **(h)** left nasal cavity 8 weeks after surgery, green arrow—healed ostium above the middle turbinate, blue arrow—middle turbinate; **(i)** left nasal cavity: lacrimal drainage irrigation test showing patency, yellow dye coming out from ostium.

Given the massive septal deviation and no visualization of the middle turbinate, an endoscopic septoplasty was performed first to create a workable space to the lacrimal sac fossa on the left. After mucosal elevation and septal cartilage/bony correction, the middle turbinate became visible and turned out to be displaced inferiorly (Fig. 2a–d).

### Step 2: EnDCR

With improved access, a transcanalicular light probe was used to identify the position of the lacrimal sac fundus endonasally. Guided by endoscopy with reference to the MR model, the lacrimal sac fossa was exposed superior to the base of the middle turbinate, followed by creation of the osteotomy and marsupialization of the sac. A mucosa-to-mucosa anastomosis was fashioned to create a large ostium. Silicone intubation of the lacrimal canaliculi was conducted with a Crawford stent. Mitomycin C was used locally (topically and as a circumostial injection) to prevent scarring and pathological granulation (Fig. 2e–i).

### Intraoperative role of MR

MR-IR provided continuous spatial orientation to the patient-specific nasal cavity geometry and adjacent anatomical landmarks throughout septoplasty and EnDCR, with particular benefit during lacrimal sac localization. The holograms were spatially anchored within the operating room adjacent to the endoscopic display and could be interactively manipulated (scaling and rotation) as required. The surgeon placed two identical holograms on either side of the endoscopy screen at different orientations to allow simultaneous viewing of the reconstruction from complementary angles. During the procedure, the hologram was manually grasped and rotated three times at critical moments when precise localization of the lacrimal sac was required. The surgeon used hand gestures to control the holographic reconstructions. MR-IR was used at the beginning of surgery to plan the septoplasty procedure and during EnDCR to support localization of the lacrimal sac. Time of MR-IR use was 10 and 30 min, respectively. Neither latency, brightness nor ergonomics issues were noted. MR did not provide tracking or navigational guidance. Endoscopic technique and light-guided localization dictated the surgical steps. This MR-IR approach is conceptually aligned with published experiences where MR enhanced intraoperative spatial cognition or planning in laparoscopic, orthopedic, spine, and maxillofacial procedures, even when not used for stereotactic navigation.^1,5,7,8,10,11^

## Postoperative Course and Outcomes

Recovery was uneventful. Postoperative examination was conducted at 2 weeks, 1 month, 2 months, and 6 months after the surgery. At the 6th-month follow-up, the patient reported complete resolution of epiphora with a Munk score = 0. Endoscopic examination showed a healed, widely patent ostium much above the middle turbinate with free flow during lacrimal irrigation. No further dacryocystitis episodes were noted.

## Discussion

In cases of secondary acquired NLDO after major facial trauma, EnDCR is challenged by severe distortion of bony and mucosal landmarks. This can compromise access, sac localization, and the orientation needed to fashion a reliable osteotomy.^6^ Our case demonstrates the technical feasibility of MR as an intraoperative *image reference* tool using an ML2 headset to maintain an accurate, patient-specific 3D anatomical model during stepwise endoscopic work. The approach complemented—rather than replaced—standard endoscopic visualization and the surgeon’s tactile perception during septoplasty and EnDCR. Several surgical fields have reported MR benefits for spatial understanding, heads-up visualization, and workflow. In laparoscopic cholecystectomy, MR holography was feasible and subjectively aided anatomy comprehension, though efficacy varied by surgeon experience.^11^ Orthopedic and maxillofacial teams have described improved planning, communication, and accurate osteotomy execution when MR was combined with or linked to navigation, with sub-millimetric or millimetric placement accuracy in selected workflows.^7,9^ Spine studies using integrated MR-supported head-mounted navigation have shown high pedicle screw accuracy. Liu et al. reported the accuracy of 205 pedicle screws consecutively placed in 28 patients with an accuracy of 98% (≈98–100% Grade A/B), underscoring that tracked systems can achieve clinical-grade stereotaxy.^10^

In the lacrimal domain, Nowak et al. recently reported VR/MR-assisted EnDCR for extremely complex nasolacrimal obstructions (congenital syndromic and post-traumatic), documenting preoperative VR planning and intraoperative MR-IR use to interrogate spatial relationships step-by-step without disrupting the endoscopic procedure. Nowak et al. used an ML2 HMD for intraoperative MR-IR support and, similar to our case, did not report any technical issues.^6^ The ML2 HMD is equipped with automatic brightness adjustment; therefore, it performs well under high illumination conditions in the operating theater before the endoscopy unit is used, as well as after the lights are dimmed. Our case aligns with this paradigm and adds a focused description of ML2-based MR-IR use in a profoundly distorted post-traumatic setting. The choice of MR assistance should match task demands and institutional resources. The MR-IR approach used in the presented case requires minimal infrastructure: a clean, high-fidelity model in the room, accessible on demand for cognitive off-loading and orientation—particularly valuable when intranasal landmarks are displaced or absent. This is distinct from surgical navigation, which depends on robust registration and tracking but can deliver quantitative instrument-target relationships; such systems have yielded excellent accuracy in spine and oral–maxillofacial work. When registration is impractical or the surgical task relies primarily on exposure and visualization (as in EnDCR), *image reference* may be the most efficient MR mode. The image guidance cannot be utilized in the endoscopic approach because overlaying a two-dimensional endoscopy screen with unregistered virtual 3-dimensional objects would obscure the surgeon’s field of view. Regulatory science emphasizes standardized evaluation of MXR devices and applications, including optical performance, latency, registration accuracy, human factors, and clinical endpoints. Adhering to emerging evaluation frameworks and reporting the MR role (image reference vs image guidance vs surgical navigation), registration approach, device model, and integration steps are critical for reproducibility. Field-level taxonomies further support consistent categorization across clinical reports.^2^

## Conclusion

This case supports the use of MR for both preoperative and intraoperative planning in the setting of severe anatomical distortion. It highlights a pragmatic adjunctive role for MR in complex lacrimal surgery alongside standard endoscopic techniques, while broader adoption will depend on continued methodological rigor and standardized reporting within medical extended reality (MXR) frameworks.

## Authors’ Contributions

M.N.: Data collection; literature review; writing—original draft. P.J.G.: Supervision; critical revision of the article for important intellectual content; final approval of the version to be published. R.N.: Conceptualization; primary surgeon; clinical management of the patient; image processing; writing—review and editing.

## Abbreviations used

3D: Three-Dimensional
CT: Computed Tomography
DCR: Dacryocystorhinostomy
DICOM: Digital Imaging and Communications in Medicine
EnDCR: Endoscopic Dacryocystorhinostomy
GLB: Type of three-dimensional file format (GL Transmission Format Binary)
HMD: Head-Mounted Display
ML: Magic Leap
ML2: Magic Leap 2
MR: Mixed Reality
MR-IR: Mixed Reality-Image Reference
MXR: Medical Extended Reality
NLDO: Nasolacrimal Duct Obstruction
SALDO: Secondary Acquired Lacrimal Duct Obstruction
VR: Virtual Reality
